# Supplementary material for: A diminutive new basilosaurid whale reveals the trajectory of the cetacean life histories during the Eocene
Source: Commun Biol. 2023 Aug 10;6:707. doi: 10.1038/s42003-023-04986-w (PMC10415296; doi:10.1038/s42003-023-04986-w)
Supplement: Supplementary file 6 — Supplementary Data 3 [file 42003_2023_4986_MOESM6_ESM.zip › Supplementary Data 3/Supplementary Data 2_BTD_ASR/trait_0056_tree.plot.pdf]

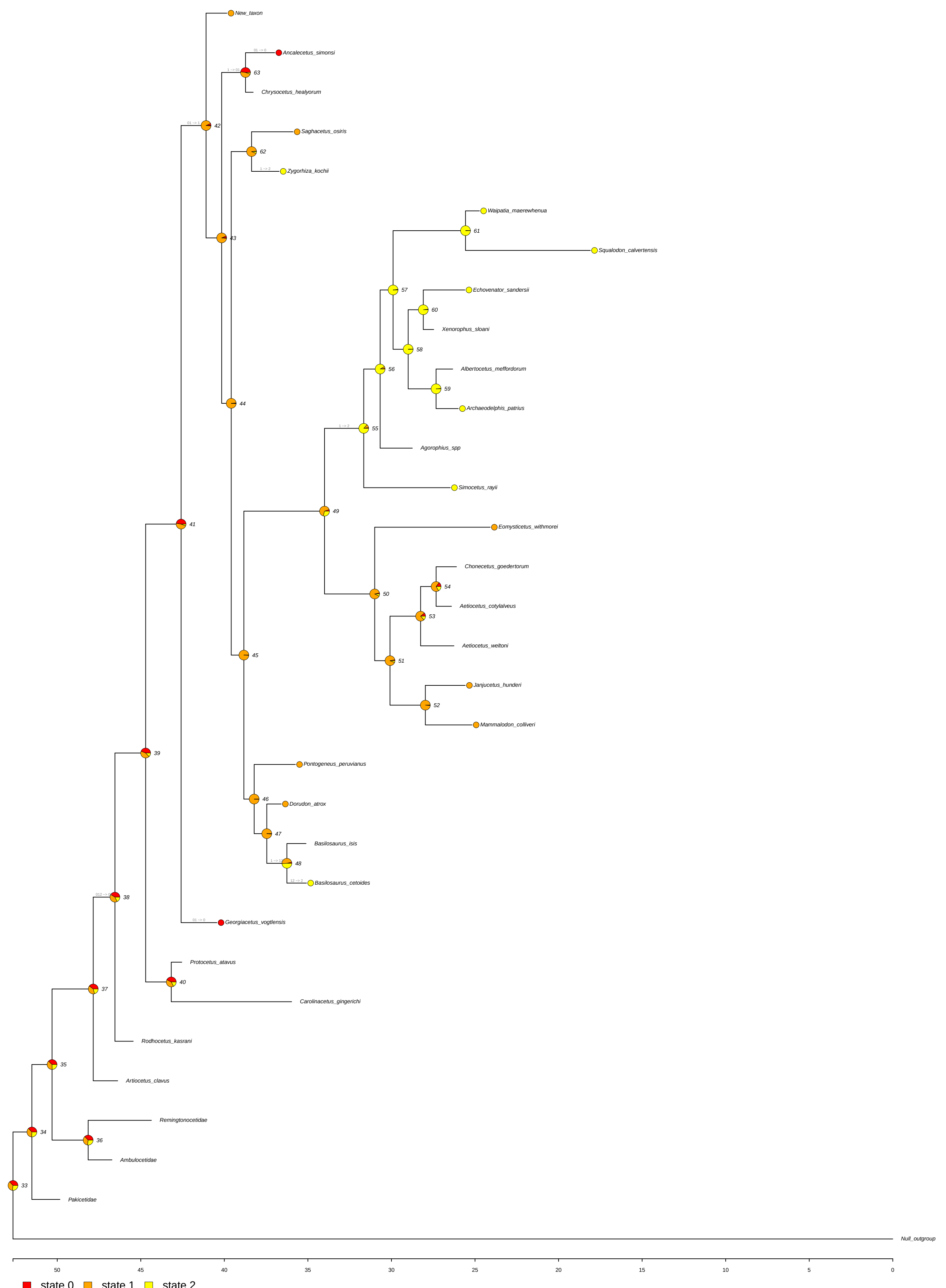
New_taxon
Ancalecetus_simonsi
Chrysocetus_healyorum
Saghacetus_osiris
Zygorhiza_kochii
Waipatia_maerewhenua
Squalodon_calvertensis
Echovenator_sandersii
Xenorophus_sloani
Albertocetus_meffordorum
Archaeodelphis_patrius
Agorophius_spp
Simocetus_rayii
Eomysticetus_withmorei
Chonecetus_goedertorum
Aetiocetus_cotylalveus
Aetiocetus_weltoni
Janjucetus_hunderi
Mammalodon_colliveri
Pontogeneus_peruvianus
Dorudon_atrox
Basilosaurus_isis
Basilosaurus_cetoides
Georgiacetus_vogtlensis
Protocetus_atavus
Carolinacetus_gingerichi
Rodhocetus_kasrani
Artiocetus_clavus
Remingtonocetidae
Ambulocetidae
Pakicetidae
Null_outgroup
50
45
40
35
30
25
20
15
10
5
0
state 0
state 1
state 2
